# Evaluation and Recommendations on Good Clinical Laboratory Practice Guidelines for Phase I–III Clinical Trials

**DOI:** 10.1371/journal.pmed.1000067

**Published:** 2009-05-05

**Authors:** Marcella Sarzotti-Kelsoe, Josephine Cox, Naana Cleland, Thomas Denny, John Hural, Leila Needham, Daniel Ozaki, Isaac R. Rodriguez-Chavez, Gwynneth Stevens, Timothy Stiles, Tony Tarragona-Fiol, Anita Simkins

**Affiliations:** 1 Duke University, Center for AIDS Research Central QA Unit, Durham, North Carolina, United States of America; 2 International AIDS Vaccine Initiative, Rockville, Maryland, United States of America; 3 Henry M. Jackson Foundation for the Advancement of Military Medicine–Division of AIDS, National Institute of Allergy and Infectious Diseases, National Institutes of Health, Department of Health and Human Services, Bethesda, Maryland, United States of America; 4 Duke Human Vaccine Institute, Center for HIV/AIDS Vaccine Immunology, Durham, North Carolina, United States of America; 5 HIV Vaccine Trials Network, Seattle, Washington, United States of America; 6 Division of AIDS, National Institute of Allergy and Infectious Diseases, National Institutes of Health, Department of Health and Human Services, Bethesda, Maryland, United States of America; 7 International AIDS Vaccine Initiative, Johannesburg, Gauteng, South Africa; 8 Qualogy Ltd, Kettering, United Kingdom; 9 International AIDS Vaccine Initiative Core Laboratory, Imperial College London, London, United Kingdom; 10 AlphaVax Human Vaccines, Research Triangle Park, North Carolina, United States of America

## Abstract

Marcella Sarzotti-Kelsoe and colleagues harmonize various approaches to Good Clinical Laboratory Practice for clinical trials into a single set of recommendations.

## Introduction

Global clinical laboratory work performed under harmonized operations is a central component for the successful conduct of phase I–III clinical trials in multiple fields of science and medicine. However, global harmonization of clinical laboratories for the analysis of specimens from clinical trials operations (i.e., for safety, diagnostic, endpoint laboratory assays) faces international challenges (e.g., laboratory logistical and technical factors), and it is subject to different interpretations of regulations and guidance materials published by the federal government, accrediting, and non-accrediting organizations (e.g., Good Laboratory Practice [GLP] [1], Clinical Laboratory Improvement Amendments [CLIA] [2], College of American Pathologists [3], International Organization for Standardization [ISO] 15189 [4], and International Conference on Harmonization [ICH] Good Clinical Practice [GCP] [5]).

In an effort to harmonize and gain consensus on international clinical laboratory operations, Good Clinical Laboratory Practice (GCLP) guidelines were originated by merging GLP and ICH-GCP principles, and were first published and copyrighted by the British Association of Research Quality Assurance (BARQA) (BARQA-GCLP) [6]. Subsequently, the Division of AIDS (DAIDS), National Institute of Allergy and Infectious Diseases (NIAID), National Institutes of Health expanded the existing knowledge on GCLP standards by publishing guidelines on GCLP (NIAID-GCLP) [7], with increased implementation guidance based on applicable portions of GLP, CLIA, the College of American Pathologists, and the International Organization for Standardization (ISO 15189). Both of these GCLP approaches were created to ensure that clinical laboratory results are reliable, repeatable, auditable, and comparable between multiple clinical laboratories. Nevertheless, differences in the implementation of GCLP by clinical laboratories have created critical inconsistencies for routine management of operations in support of clinical trials and have caused an urgent need to clarify and harmonize four central GCLP elements for optimal management and clinical laboratory operations. These GCLP elements—discussed in this paper—are training, auditing, assay validation, and proficiency testing.

The differences regarding the implementation of universal standards of GCLP for clinical laboratory operations (i.e., clinical laboratories performing safety, diagnostic, and endpoint assays) in the conduct of clinical trials have been experienced in the HIV study field. However, it is expected that this problem will have broader implications in clinical trials, involving multiple fields. This paper addresses for the first time an attempt to harmonize these GCLP approaches into a single set of recommendations for optimal operations and management that can be followed by clinical laboratories, not only in the HIV field, but also possibly in other science and medical fields.

## Background

The Global HIV Vaccine Enterprise (GHAVE) [8] created an alliance of independent organizations around the world dedicated to the development of a preventive HIV vaccine, spanning vaccine discovery, product development, manufacturing, and clinical trials. Both the International AIDS Vaccine Initiative (IAVI) and DAIDS are globally recognized organizations and work collaboratively in clinical trials under GHAVE. In the HIV field, clinical laboratory standardization based on GCLP compliance is one of GHAVE's primary goals. Currently, within GHAVE there are two approaches on how to achieve GCLP compliance in a clinical laboratory environment. The first approach is followed by IAVI, and it is based on BARQA-GCLP [6],[9],[10]. The second approach is followed by DAIDS, and it is based on NIAID-GCLP [7]. The two approaches cover the same general core elements [6],[7], as listed in Table 1: BARQA-GCLP and NIAID-GCLP Core Elements.

**Table 1:** BARQA-GCLP and NIAID-GCLP core elements.

BARQA-GCLP	NIAID-GCLP
Organization and personnel	Organization and personnel
Facilities	Physical facilities
Equipment, materials, and reagents	Equipment, test, and control
Standard operating procedures	Testing facility operations
Planning, conduct, and reporting	Specimen transport and management; laboratory information system; verification of performance; records and reports; personnel safety
Quality audit	Quality management
Quality control	See: Test and control
Storage and retention of records	See: Records and reports
Confidentiality	See: Records and reports

The BARQA-GCLP guidelines were written in response to the global adoption of the GCP guidelines to provide a framework to organizations that undertake laboratory analysis of specimens from clinical trials, on the facilities, systems, and procedures that should be present to ensure the reliability, quality, and integrity of the work, and to ensure that results are generated and reported to satisfy GCP expectations. The BARQA-GCLP guidelines were written purposely in a generic format to allow for sponsor interpretation and implementation, but to meet the global challenge of GCP compliance.

NIAID, as a sponsor of multiple HIV clinical trials, developed the NIAID-GCLP guidelines with the objective of providing a single unified document that encompasses sponsor requirements and that embraces regulatory and guidance materials to guide the conduct of clinical laboratory testing for human clinical trials. The NIAID-GCLP is recognized as the minimum clinical laboratory operation requirements to participate in DAIDS-sponsored clinical trials.

Although both the BARQA-GCLP and the NIAID-GCLP guidelines embrace clinical laboratories conducting safety, diagnostic, and endpoint assays in their distinctive approaches, they differ in four critical GCLP elements for optimal management of clinical laboratory operations: training, auditing, assay validation, and proficiency testing. The latter four GCLP elements were selected as they represent paramount stages in the conduct of GCLP-compliant clinical studies supported by clinical laboratories: from the general set-up (training of personnel and assay validation) through the more specific elements of assay conduction and laboratory oversight (audits, proficiency testing, and accreditation). Briefly, key issues pertaining to the four discrepant GCLP elements according to the BARQA-GCLP and the NIAID-GCLP guidelines are the following: (1) Training: since training for GCLP is provided by different organizations following BARQA-GCLP and NIAID-GCLP, it is crucial to define what constitutes training within the GCLP boundaries. (2) Auditing: the BARQA-GCLP guidelines support a systems approach to auditing, do not require an audit checklist, and endorse laboratory accreditation for GCLP compliance. In contrast, the NIAID-GCLP guidelines follow a compliance approach to auditing, require an audit checklist, and do not endorse laboratory accreditation for GCLP compliance. (3) Assay validation: validating an assay consists of evaluating the applicability of the parameters described in the ICH Q2A [11] and Q2 (R1) [12] guidelines for relevance to an assay and its intended use. The BARQA-GCLP guidelines are very generic on the topic of assay validation, while the NIAID-GCLP guidelines provide very detailed requirements in this matter. (4) Proficiency testing: NIAID-GCLP guidelines describe a proficiency testing program as a required external quality assurance program. The BARQA-GCLP guidelines only mention that a laboratory should subscribe to external proficiency schemes to demonstrate the competency of the work performed.

Currently, clinical trial sponsors in the HIV study field are requiring clinical laboratories to adhere to GCLP guidelines to optimize laboratory operations and to strive for consistency and integrity of results across multiple sites. In the HIV field, pluripotent clinical laboratories are faced with the following question: which of the two main GCLP (BARQA-GCLP or NIAID-GCLP) approaches should be followed to become GCLP compliant, meet GCLP expectations from different sponsors, and obtain consistent laboratory results across trials and sites? These clinical laboratories must have minimum and common levels of standardization within the interpretation and implementation of GCLP, so that consistent and reliable results are obtained in support of clinical trials and can be submitted to regulatory health authorities. As experienced in the HIV field, clinical laboratories supporting clinical trials in other fields of science and medicine are facing a similar situation [13].

## Purpose and Scope

The purpose of this paper is to propose a harmonized interpretation of the four GCLP critical elements listed above, for optimal management of clinical laboratory operations, which are critical for successful conduction of clinical trials. These GCLP elements were selected upon reviewing inconsistencies in the BARQA-GCLP and the NIAID-GCLP guidelines, which have affected the harmonization of clinical laboratory operations. The ultimate goal of this paper is to raise public awareness on the need to harmonize GCLP for clinical laboratory operations to optimally support clinical trials, and to call on the attention of the United States Food and Drug Administration, the Organization for Economic Co-operation and Development, the European Medicines Agency, and all other regulatory agencies worldwide to consider the information presented here, so that universal GCLP standards are developed. This paper was originated from a group of authors with expertise in phase I–III HIV clinical trials, GCLP, and regulatory materials such as GLP [14] and GCP, who convened the “Workshop on GCLP Guidelines for International Clinical Trials: Interpretation and Implementation” (October 9–10, 2007, in Raleigh, North Carolina [15]), but its scope extends to other study fields, such as cancer, malaria, tuberculosis, stem cell, or other therapeutic interventions.

## GCLP Recommendations

This section consists of the GCLP recommendations agreed upon by the authors during the Workshop and from follow-up sessions.

### Training

The sponsor of a clinical trial or study is responsible for ensuring that all individuals working on or supporting a clinical trial have the appropriate experience or training to perform their functions. Laboratory management is responsible for developing minimum requirements for training per their site's experience and capability. Training of laboratory and support staff should have two main constituents: (1) training to perform the job and (2) training for in-study protocol requirements. Each laboratory conducting safety, diagnostic, and endpoint assays should have a documented and ongoing training program and routine assessment of competence in the performance of the individual's role. Each site should maintain individual training records for all staff members, which confirm the training received and the level of competency attained.

GCLP training is recommended for any member of the laboratory staff involved in work that supports a clinical trial. At a minimum, the clinical laboratory manager must be appropriately trained and qualified to perform his/her role and have received GCLP training. Similarly, staff working within the clinical laboratory should be suitably trained and qualified in those parts of GCLP applicable to the work they perform.

There are different GCLP trainings offered by different organizations following BARQA-GCLP or NIAID-GCLP, and the training effectiveness may vary based on the experience or skill of the trainer. GCLP training should be provided by a recognized institution/accrediting body or experienced trainer. A GCLP trainer must have an in-depth understanding of GCLP gained by working in the environment and must be experienced and proficient at educating and competent in communicating to a trainee the requirements of GCLP. It is recommended that laboratory management verify the credentials of a trainer or an organization before obtaining GCLP training.

### Auditing

Periodic audits of clinical laboratories involved in the conduct of a clinical trial should be performed. The frequency of audits is determined based on sponsors' requirements and specific clinical laboratory needs to meet GCLP. GCLP sponsor audits of a clinical laboratory should follow a sponsor-approved master audit plan that covers the elements of GCLP to ensure compliance with the appropriate regulations, protocols, standard operating procedures, and analytical plans. A generic example is provided in Text S1: Example of a GCLP Master Audit Plan.

It is recommended that an audit checklist be used as a tool to assist auditors in consistently reviewing categories and specific processes applicable to each clinical laboratory site within a clinical trial and to document progress over time. An example of a checklist is provided in Text S2: Example of an Audit Checklist.

An audit is a sponsor-driven laboratory (e.g., clinical laboratory) assessment related to a trial or study; as opposed to GCLP accreditation, which is an independent assessment of a laboratory (e.g., clinical laboratory) to operate in accordance with GCLP irrespective of the sponsor of a trial or study. Although one accrediting body should give confidence to any sponsor that a facility operates following GCLP standards, GCLP accreditation is not yet globally provided by regulatory agencies and there is no governing body overseeing the accreditation process. The latter represents a gap in the GCLP field to be addressed by regulatory agencies worldwide. Qualogy, an independent organization, currently provides GCLP accreditation, endorsed by IAVI. A Qualogy GCLP-accredited clinical laboratory is assessed for compliance annually for the first three years, and every two years thereafter. In contrast, NIAID does not endorse accreditation for its sponsored laboratories, but provides guidance for GCLP compliance on a continuous and as needed basis. Likewise, NIAID-sponsored laboratories are assessed for GCLP compliance with a frequency that is determined by the sponsor and based on the specific laboratory needs. The frequency of GCLP laboratory assessment can be as minimal as once per year. While consensus was not reached on the GCLP accreditation requirement, the authors did agree on the need for external auditing of laboratories for GCLP compliance and that laboratories that have obtained accreditation status should still perform internal quality assurance audits. The lack of consensus on GCLP accreditation requirement reflects the technical gap existing in the GCLP field that needs to be addressed by regulatory health authorities worldwide; an evolution of the GCLP field that is out of the scope of the present paper. Additionally, the authors agree that the management of a clinical laboratory must evaluate the risks and costs associated with obtaining or not obtaining GCLP accreditation. The authors wish to raise awareness on the issue of GCLP accreditation and the need to establish a global accrediting body.

### Assay Validation

All methods used for safety or endpoint analyses in clinical trials need to be appropriately validated and demonstrably fit for purpose. Methods defined as waived by CLIA, or methods introduced prior to April 2003, do not require validation [2]. For non-waived CLIA assays, such as immunogenicity endpoint assays, appropriate ICH Q2A and Q2 (R1) guidelines [11],[12] for assay validation should be used by clinical trial laboratories to demonstrate fitness for purpose of the assay in question [16]–[19]. Validating an assay consists of evaluating the applicability of the parameters described in the ICH Q2 guidelines for relevance to the assay and its intended use: accuracy, precision, limit of detection, limit of quantitation, specificity, linearity and range, ruggedness/robustness, and system suitability. However, it is at the discretion of the validating clinical laboratory conducting endpoint assays to define, based on statistical analysis, the critical parameters that are necessary to validate an assay. For example, if a “gold standard” assay does not exist for measuring the analyte in question, accuracy, as defined by the guidance document, cannot be addressed, as it is a specific comparison to a “gold standard” assay. This does not preclude one from validating the assay for other parameters such as precision, linearity, limits of detection, and quantitation. The process of assay validation involves qualification of the assay and the establishment of pass/fail criteria to be used for final assay validation. By no means should simple intra- and inter-laboratory comparison, and/or the use of positive/negative controls for an assay, be considered assay validation. Finally, in the absence of a generalized proficiency testing program, a validated assay can be transferred to another clinical laboratory through demonstration of concordance with the clinical laboratory in which the assay was validated or another that has successfully transferred the assay.

### Proficiency Testing

A proficiency testing program consists of an evaluation of data, provided by multiple clinical laboratories using assays capable of identifying the same analyte or diagnostic outcome on the same set of samples, by a central unit that statistically analyzes the data. The analysis determines the performance of each individual laboratory compared to the others or, when available, to a “gold standard.” Proficiency testing programs for many of the routine hematology and biochemistry assays do exist, and clinical laboratories should participate in these programs, where applicable.

A proficiency testing program for a new clinical endpoint assay should be based on the detection and/or quantitation of specific analytes under predetermined laboratory conditions and procedures and should be provided on a routine basis [20]–[25]. The proficiency program should consist of the following, when possible: (1) A standardized set of specimens that contain the analytes to be detected. These analytes should be tested by a large enough sampling of laboratories to establish a statistically relevant mean (“gold standard”) of the results measured under the proficiency testing. (2) A defined kit or a set of reagents, materials, and equipment that should be available to all participating laboratories. (3) A set of general and assay-specific instructions for the conduct of the proficiency testing. The general instructions should also include the defined criteria of outcome acceptability based on its statistical similarity/dissimilarity to the mean results of the assays being assessed by all participating laboratories. A reporting structure should exist to inform laboratories if their results are within the acceptable range. Results that are outside of the established range (outliers) should be investigated to determine if improved protocols resulted in better detection of the analyte while maintaining a low background and false positive rate. (4) Frequency of proficiency testing will be determined based on the inherent variability and complexity of the assay being tested, laboratory needs, and sponsor requirements.

For many immunogenicity assays, there is no “gold standard” currently available, and participants' results will establish the “gold standard” value over time. The larger the number of participating laboratories, and the greater the frequency of the proficiency testing, the greater the confidence will be in generating the relevant mean. Irrespective of the existence of a proficiency scheme, GCLP-compliant laboratories should regularly evaluate the performance of assays for accuracy and precision through trend analysis of a consistent positive control. Each individual laboratory should determine the frequency of internal laboratory testing, based on their specific needs.

Pass/fail criteria for a proficiency testing program is based on the statistical similarity of the results of an individual laboratory when compared to the mean of the results of all participating laboratories. Therefore, it is important that proficiency testing reports include summary statistics and graphical presentations of performance of each laboratory along with the mean of results obtained by all participating laboratories. Performance testing is not about passing or failing; it is about identifying a drift of an assay and the need to take actions to investigate the causes of the drift and to correct them for consistency of results over time and across multiple laboratories. As an example, in the performance testing of immunological assays, a coefficient of variation of less than 30% for cell-based assays and ∼10% for humoral (ELISA)-based assays is considered acceptable [26].

## Benefits

Standardized GCLP requirements would benefit clinical laboratories conducting safety, diagnostic, and endpoint assays in support of clinical trials and would potentially benefit other laboratories globally, both by providing a consistent direction in GCLP compliance, and by allowing laboratories to put into practice a unified set of GCLP-defined procedures that will enhance reproducibility and reliability of results. The latter is especially critical when conducting trials that use multiple international laboratory sites.

## Conclusions

Harmonization exists among global organizations as to the need for a quality system in clinical laboratories that analyze specimens from clinical trials. The authors reached a consensus that GCLP compliance is the minimal requirement that clinical laboratories should meet to increase adherence to standardized practices and procedures, optimize management operations of clinical laboratories, and enhance obtaining reproducible and reliable results, while ensuring the safety of human research participants.

General consensus on three out of the four selected GCLP elements was reached and presented as GCLP recommendations of this paper. The authors recognize that individual study sponsors may apply sponsor-specific standards for the implementation of GCLP elements across different areas of their studies and that sufficient flexibility exists in how compliance with such elements should be met. One such example may be the frequency of GCLP training for the clinical laboratory staff: it was agreed that staff should receive GCLP training, but the frequency of such training will be decided by the clinical laboratory management and the study sponsor. The GCLP element regarding clinical laboratory accreditation for GCLP compliance remains open, since at the present time there is no international, universal, and publicly available accrediting organization acceptable to all parties involved in clinical trials worldwide. With time, and as the GCLP standard becomes globally accepted, GCLP accreditation will find a place in the implementation of GCLP for laboratories. It is the authors' wish to raise awareness on the issue of GCLP accreditation and on the need to establish a global accrediting body.

## Supporting Information

Text S1 Example of a GCLP master audit plan.
(0.08 MB PDF)

Text S2 Example of an audit checklist.
(0.19 MB DOC)

## Abbreviations

BARQA: British Association of Research Quality Assurance
CLIA: Clinical Laboratory Improvement Amendments
DAIDS: Division of AIDS
GCLP: Good Clinical Laboratory Practice
GCP: Good Clinical Practice
GHAVE: Global HIV Vaccine Enterprise
GLP: Good Laboratory Practice
IAVI: International AIDS Vaccine Initiative
ICH: International Conference on Harmonization
NIAID: National Institute of Allergy and Infectious Diseases
