# Supplementary material for: Evaluation and Recommendations on Good Clinical Laboratory Practice Guidelines for Phase I–III Clinical Trials
Source: PLoS Med. 2009 May 5;6(5):e1000067. doi: 10.1371/journal.pmed.1000067 (PMC2670502; doi:10.1371/journal.pmed.1000067)
Supplement: Text S1 — Example of a GCLP master audit plan. (0.08 MB PDF) [file pmed.1000067.s001.pdf]

SUPPLEMENTARY APPENDIX A: Example of a GCLP Master Audit Plan

Laboratory Site Information	
**Laboratory Site Identification**	
**Laboratory Manager**	
**Address of Laboratory Site**	

Study Protocol Title and No.: *[Enter information, if applicable]*

Audit scheduled for: *[Enter Tentative Dates]*

Purpose of Audit
This clinical laboratory was chosen to be audited *[add reason for the audit].*
Examples:

1. Assess a lab's ability to conduct lab operations in accordance with GCLP guidelines/standards and other regulations
2. Ensure lab continues to meet applicable regulations/guidelines/standards and sponsor requirements
3. For cause audits to assess/identify areas of concern at the clinical lab

This audit will be performed as per *[add SOP number or regulation]*.

Scope of the Audit:
The audit will follow a sponsor approved audit checklist. *[Delete if not applicable]*
This audit will consist of the following *[Add or subtract as needed]:*

- Tour the facilities
- Evaluate the facility for the construction and size suitability
- Evaluate the flow of the working practices applicable to the facility
- Interview the Laboratory Management and other key staff regarding their role and responsibilities (Organization and Personnel).
- Evaluation of sites' level of compliance with the study protocol requirements, Standard Operating Procedures, and adherence to applicable regulations and GCLP guidelines
- Review of the Quality Assurance program
- Evaluation of documentation practices
- Review of a representative sample of the laboratory data
  Need to define the data sampling plan for the audit
  [Example: % of data to review]
- Review assay acceptance and repeat process
- Review reagent preparation and qualification
- Review of the assay method validation
- Review receipt, storage and retention of clinical samples
- Evaluation of suppliers or vendor qualification
- Evaluation of record storage conditions, retention and archive process
- Evaluation of equipment calibration/verification, maintenance and use
- Evaluation of training documentation

- Evaluation of laboratory compliance to safety regulations
- Evaluation of Computer Systems
- Evaluation of the Laboratory Management

Reporting of Findings:

A. The audit report will be circulated for review within *[add number of days]* business days after the completion of the audit to the following individuals:
    - *[List Names and Titles]*

B. The audit report will be distributed to the *[title of responsible individual]* for response and corrective action completion. The auditor(s) or Sponsor will review the audit report responses instructions with *[title of responsible individual]*. Responses and corrective actions should be returned to the auditor or Sponsor within *[add number of days]* business days of the receipt of the audit report. If the *[title of responsible individual]* is not able to respond to the audit findings within the stated timeframe, *[title of responsible individual]* must submit a written extension request with an anticipated completion date to the auditor or Sponsor.

C. The audit report, audit responses and corrective actions will be circulated for review at the closure of the audit process to the following:
    - *[List Names and titles]*

Signatures below indicate review and approval of this Audit Plan

________________________________ ________________

[Name of auditor and Title] Date

________________________________ ________________

[Sponsor Name/Title] Date

________________________________ ________________

[Sponsor Name/Title] Date
