# Supplementary material for: Evaluation and Recommendations on Good Clinical Laboratory Practice Guidelines for Phase I–III Clinical Trials
Source: PLoS Med. 2009 May 5;6(5):e1000067. doi: 10.1371/journal.pmed.1000067 (PMC2670502; doi:10.1371/journal.pmed.1000067)
Supplement: Text S2 — Example of an audit checklist. (0.19 MB DOC) [file pmed.1000067.s002.doc]

**Text S2: Example of an Audit Checklist**

| **Organization and Personnel** | **Yes** | **No** | **NA** | **Observations/Recommendations** |
| --- | --- | --- | --- | --- |
| Organizational chart exists and accurately represents the organization? |  |  |  |  |
| Is the laboratory affiliated with other organizations?  Identify the organizations: |  |  |  |  |
| Are training records available?  List the components of the training record: |  |  |  |  |
| Are there personnel curricula (training matrix/plan) established and documented for each individual? |  |  |  |  |
| Does the training program include new hire training and re-qualification training for personnel? |  |  |  |  |
| Has personnel been appropriately trained to perform functions required by job descriptions? |  |  |  |  |
| Is there a procedure to assess and document personnel competency on an annual basis? |  |  |  |  |
| Is there an escalation process by which personnel that do not pass competency are retrained etc.? |  |  |  |  |
| Has the Laboratory manager received GCLP training? If so, by what organization and when? |  |  |  |  |
| Has personnel received regulatory training?  GCLP  GCP  GLP  Other: |  |  |  |  |
| Has personnel received health/safety training?  List safety training provided: |  |  |  |  |
| Is there a system in place for personnel to report any safety concerns or incidents? |  |  |  |  |
| Does the laboratory have sufficient qualified personnel to perform functions that support the GCLP clinical trial? |  |  |  |  |
| Is there a list of consultants and is their qualifications maintained? |  |  |  |  |
| Are external contractors/vendors utilized? Are they qualified/approved for use?  Is there an SOP that outlines this process? |  |  |  |  |
| Is there a Quality Assurance Unit? If so, what are the roles of the Quality Control and the Quality Assurance group? |  |  |  |  |
| Does the Quality Assurance Unit perform audits, trend metrics and report the results to the Laboratory Management? |  |  |  |  |
| Is the Quality Assurance Unit independent from the personnel engaged in the direction or conduct of a clinical trial? |  |  |  |  |
| **Certifications/Licenses** | **Yes** | **No** | **NA** | **Observations/Recommendations** |
| Does the laboratory maintain any certifications/licenses?  CAP  Date:  CLIA  Date:  Other: |  |  |  |  |
| Are there copies of certifications/licenses available? |  |  |  |  |
| **Standard Operating Procedures/Methods** | **Yes** | **No** | **NA** | **Observations/Recommendations** |
| Is there a governing SOP that outlines the creation, revision, approval, distribution, document control and retirement of SOPs? |  |  |  |  |
| Are SOPs in compliance with the current version governing SOPs? |  |  |  |  |
| Is there a current index listing of the SOPs available? |  |  |  |  |
| Is there a schedule for review of the SOPs? |  |  |  |  |
| Are the SOPs in locations where they are used? |  |  |  |  |
| Is there a system for documenting and handling SOP/method deviations and CAPAs? |  |  |  |  |
| Is there a change control system for SOP/Methods? |  |  |  |  |
| **Facility** | **Yes** | **No** | **NA** | **Observations/Recommendations** |
| Security and confidentiality is adequate to prevent unauthorized access to records/ test samples and a procedure to report unauthorized access exists? |  |  |  |  |
| Is there sufficient space to store materials, archive records, equipment to function properly and conduct laboratory testing? |  |  |  |  |
| Is the work flow designed to prevent contaminations and mix-ups of test samples |  |  |  |  |
| Is the facility maintained and clean? |  |  |  |  |
| Is there safety equipment (e.g. showers. eyewash stations) available? Is the equipment maintained? |  |  |  |  |
| Are updated Material Safety Data sheets and Certificate of Analysis available? |  |  |  |  |
| Are there environmental controls within laboratory and are the controls monitored? |  |  |  |  |
| Are personnel wearing appropriate garmenting for designated areas? |  |  |  |  |
| Is there an SOP detailing the designated routes and methods available for waste disposal? |  |  |  |  |
| Is the biohazardous and hazardous chemical waste disposal described? |  |  |  |  |
| Is there a sanitation or cleaning procedure established and is being followed and documented? |  |  |  |  |
| Are facilities maintenance procedures established and being followed and documented? |  |  |  |  |
| Does the Laboratory have a disaster recovery plan that covers all areas of the facility including computer systems and equipment? |  |  |  |  |
| Are generators utilized at the facility? |  |  |  |  |
| Does the lab have an SOP for the testing and maintenance of generators? Request to review generator logs. |  |  |  |  |
| Does the lab use a water purifying system?  Are there logs to show maintenance of the system?  Grade of water utilized? |  |  |  |  |
| **Equipment** | **Yes** | **No** | **NA** | **Observations/Recommendations** |
| Is equipment used for GCLP studies readily distinguishable from equipment used for non-GCLP Studies? |  |  |  |  |
| Is there a Master Equipment Inventory present? |  |  |  |  |
| Are there site-specific SOPs detailing equipment use, maintenance and calibration? |  |  |  |  |
| Is the equipment utilized in the lab suitable to perform the required operations? |  |  |  |  |
| Are user logs utilized for equipment and do they include a chronological record of use? |  |  |  |  |
| Log entries show the date, time, name of person performing and checking the work, as appropriate. |  |  |  |  |
| Equipment calibration and/or preventative maintenance schedules have been established and are being followed and documented |  |  |  |  |
| Records of equipment calibration or maintenance are maintained in the laboratory and archived. |  |  |  |  |
| Is there a system for moving or removing a piece of equipment from service or tagging the equipment? Documentation? |  |  |  |  |
| Calibration of the equipment is traceable to NIST or another recognized standards institution |  |  |  |  |
| Are there established tolerance limits for the equipment?  Who established the tolerance ranges? |  |  |  |  |
| Are the equipment manuals available? |  |  |  |  |
| Is there a written equipment qualification/validation program? |  |  |  |  |
| **Laboratory Controls** | **Yes** | **No** | **NA** | **Observations/Recommendations** |
| Are there an assay validation, re-validation and limited validation process outlined in a SOP? |  |  |  |  |
| Is there a written procedure for repeat testing or invalidating lab data? Is there a repeat decision tree? |  |  |  |  |
| How are results that fail specifications investigated or non-conformances investigated? |  |  |  |  |
| Are there validated methods and acceptance criteria for each test method? |  |  |  |  |
| Is there a SOP for significant figures? |  |  |  |  |
| Is there a SOP that outlines good documentation practices? |  |  |  |  |
| **Reagent and Solution Labeling and Qualification** | **Yes** | **No** | **NA** | **Observations/Recommendations** |
| Is there an SOP that outlines how reagents are labeled, how expiration dates are established? |  |  |  |  |
| Are reagents qualified for use? Is parallel testing of reagents performed? |  |  |  |  |
| Is there a current inventory of all reagents and solutions? |  |  |  |  |
| **Sample Shipment, Receipt and Storage** | **Yes** | **No** | **NA** | **Observations/Recommendations** |
| Is there a SOP for sample receipt, shipment and storage of materials and test samples? |  |  |  |  |
| Does the SOP contain a chain of custody procedure? |  |  |  |  |
| Is the sample receipt area maintained separate from the sample processing area? |  |  |  |  |
| **Data handling Procedures and Computer**  **Validation** | **Yes** | **No** | **NA** | **Observations/Recommendations** |
| Is access to computers limited by an individual username and password system (lab members cannot share a username)? |  |  |  |  |
| How is the computer network and computer systems maintained, if applicable? |  |  |  |  |
| Are there a computer validation master plan and/or SOPs? |  |  |  |  |
| List computers systems and software utilized. Validated? |  |  |  |  |
| Are changes to computer systems controlled and documented? |  |  |  |  |
| Are records of computer system errors maintained and investigated? |  |  |  |  |
| Are records of hardware maintenance and repairs maintained? |  |  |  |  |
| Are computers backed up routinely to prevent loss of data? Is there a back up log? |  |  |  |  |
| Is there a preventative maintenance program for computer systems? |  |  |  |  |
| **Records and Reports** | **Yes** | **No** | **NA** | **Observations/Recommendations** |
| A documentation control system exists and is functional. |  |  |  |  |
| Is raw laboratory data recorded in lab notebooks, electronically, or controlled data sheets? |  |  |  |  |
| Are laboratory final reports generated for clinical studies?  Who reviews the reports? |  |  |  |  |
| Is there a SOP that outlines the content of the final report? |  |  |  |  |
| Is there a SOP or a system for the retention, storage, and destruction of records? |  |  |  |  |
| How does the site ensure the sponsor’s proprietary information is not disclosed to unauthorized personnel or external organizations? |  |  |  |  |
| **Record Retention and Archival** | **Yes** | **No** | **NA** | **Observations/Recommendations** |
| Is there a dedicated facility/area for the archival of records? |  |  |  |  |
| Is there control access to the archival facility? |  |  |  |  |
| Is the environment of the facility monitored and controlled? |  |  |  |  |
| Is the procedure for archiving records outlined in an SOP? |  |  |  |  |
| Is the retention time for records stated in the SOP? |  |  |  |  |
| Is there a method of electronic data archive? |  |  |  |  |
